# Septic Cardiac Remodeling: A New Concept in Cardiac Dysfunction Induced by Experimental Sepsis

**DOI:** 10.3390/antiox15050630

**Published:** 2026-05-15

**Authors:** Nayane Maria Vieira, Letycia Netto de Paula Cunha, Carolina Rodrigues Tonon, Marina Gaiato Monte, Paola da Silva Ballin, Natália Fernanda Ferreira, Dijon Henrique Salomé de Campos, Camila Renata Correa, Gilson Masahiro Murata, Paulo Eduardo Martins Ribolla, Diego Peres Alonso, Taline Lazzarin, Paula Schmidt Azevedo, Bertha Furlan Polegato, Sergio Alberto Rupp de Paiva, Marina Politi Okoshi, Katashi Okoshi, Camila Molina Soares, Maria Cláudia Irigoyen, Marcos Ferreira Minicucci, Leonardo Zornoff

**Affiliations:** 1 Department of Internal Medicine, Botucatu Medical School, São Paulo State University—UNESP, Botucatu 18618687, Brazilcarolina.tonon@unesp.br (C.R.T.); marcos.minicucci@unesp.br (M.F.M.); leonardo.zornoff@unesp.br (L.Z.); 2 Department of Pathology, Botucatu Medical School, São Paulo State University—UNESP, Botucatu 18618687, Brazil; 3 Laboratory of Cellular, Genetic, and Molecular Nephrology, Renal Division, Medical School, University of São Paulo, São Paulo 01246903, Brazil; 4 Biotechnology Institute (IBTEC) & Biosciences Institute at Botucatu (IBB), São Paulo State University—UNESP, Botucatu 18618687, Brazil; 5 Experimental Laboratory of Hypertension, Heart Institute (InCor), University of São Paulo (USP), São Paulo 05508900, Brazil

**Keywords:** septic cardiomyopathy, cardiac remodeling, fibrosis, gut microbiota

## Abstract

Septic cardiomyopathy is recognized as an acute, transient, and reversible condition. However, septic insult may induce latent changes characteristic of cardiac remodeling, with future consequences. Therefore, the present study aimed to evaluate the morphological and functional cardiac changes in the acute and subacute phases (with 7-day follow-up) in male Wistar rats subjected to experimental sepsis using a cecal ligation and puncture (CLP) model. In the acute phase, the animals underwent echocardiographic assessment at baseline and 48 h after the induction of sepsis. In the subacute 7 days follow-up, animals were allocated in control and sepsis groups. After this period, the animals underwent echocardiographic assessment, followed by euthanasia, papillary muscle testing, and subsequent morphometric and biochemical analyses. Fecal samples from six animals per group were collected at baseline and after 7 days for microbiota analysis. In the acute phase, echocardiographic assessment revealed that, following sepsis, animals exhibited reduced systolic function. In the subacute 7 days follow-up, both echocardiogram and papillary muscles revealed cardiac dysfunction in the sepsis group. Cardiomyocyte cross-sectional area and collagen content were significantly greater in the sepsis group compared with that in the control group. Analysis of maximal enzymatic activities involved in cardiac energy metabolism and oxidative stress biomarkers revealed no significant differences between groups. Considering microbiota assessment, beta diversity analysis revealed significant differences between septic animals and controls. In conclusion, sepsis was associated with persistent systolic/diastolic dysfunction, cardiomyocyte hypertrophy, and fibrosis after 7 days. These data suggest that septic cardiomyopathy should not be considered merely an acute, transient, and reversible condition in this experimental context.

## 1. Introduction

Pathologic cardiac remodeling plays a critical role in the initiation and progression of cardiac dysfunction induced by various stimuli. However, a significant proportion of cases exhibit reverse remodeling, characterized by the restoration of cardiac myocyte size and left ventricular (LV) chamber geometry, resulting in improvement or normalization of cardiac function [1].

In the specific context of ejection fraction (EF), this condition is termed heart failure with improved ejection fraction (HFimpEF). Despite LVEF recovery, patients with HFimpEF remain at risk for adverse outcomes because improvement in LVEF does not indicate complete myocardial normalization. Persistent dysregulation of cardiac myocytes and alterations in the extracellular matrix are typically observed. Therefore, HFimpEF represents a state of remission rather than recovery, with patients remaining susceptible to recurrent LV dysfunction when exposed to various stressors [1]. This phenomenon has important clinical implications, particularly in the context of sepsis.

Sepsis may also be associated with myocardial dysfunction, a condition known as septic cardiomyopathy. This disorder is associated with significantly high mortality. Despite its clinical importance, effective therapeutic options for septic cardiomyopathy remain limited, largely because of an incomplete understanding of its underlying pathogenesis [2,3].

Over the past few decades, research in both animal models and human studies has identified multiple pathogenic mechanisms of septic cardiomyopathy, although many aspects remain incompletely understood [4,5,6]. Initially, this condition was thought to be primarily induced and modulated by inflammatory cytokines. However, more recent studies have emphasized additional mechanisms, including excessive nitric oxide production, mitochondrial dysfunction, and oxidative stress, as contributing factors in septic cardiomyopathy [7,8]. Importantly, this condition was studied mainly in the acute phase of sepsis.

Although the precise definition of septic heart disease remains challenging, it is generally regarded as an acute, transient, and reversible condition [9,10,11]. In this sense, pathologic cardiac remodeling plays a critical role in the initiation and progression of cardiac dysfunction induced by various stimuli. However, a significant proportion of cases exhibit reverse remodeling, characterized by the restoration of cardiac myocyte size and left ventricular (LV) chamber geometry, resulting in improvement or normalization of cardiac function [12]. However, septic insult to the heart may induce latent alterations characteristic of cardiac remodeling, with future consequences. Therefore, this study aimed to evaluate cardiac morphological and functional changes during the acute and subacute phases (with 7-day follow-up) in male Wistar rats subjected to experimental sepsis by CLP model.

## 2. Materials and Methods

### 2.1. Sepsis Phases

#### 2.1.1. Acute Cardiac Dysfunction Induced by Sepsis

This study was approved by the Committee for Experimental Research Ethics of the Botucatu Medical School, UNESP (protocol no. 1348/2020).

The animals were obtained from the Central Bioterium of the Botucatu Medical School (Botucatu, São Paulo, Brazil). The rats were housed in groups of up to four animals per cage with free access to water and maintained under a 12 h light/dark cycle at 25 °C and controlled humidity. Environmental enrichment was provided using paper balls and polyvinyl chloride tubes.

After an acclimatization period, the animals (n = 20) underwent baseline echocardiographic assessment (T0). Sepsis was then induced by cecal ligation and puncture, as described by Chaudry et al. [13] and adapted by our group. This CLP model is widely recognized for reproducing the major hemodynamic, metabolic, and immunological phases observed in human sepsis [2,14].

The animals were anesthetized with ketamine (70 mg/kg, intramuscular [i.m.]) and xylazine (5 mg/kg, i.m.), followed by trichotomy and abdominal disinfection with 70% alcohol. The cecum was ligated 2 cm from the ileocecal valve using 5-0 monofilament suture (TECHNOFIO) and a 22G needle to avoid necrosis. It was then punctured with the same needle and gently compressed to extrude fecal content before being repositioned. The peritoneum was closed with 3-0 silk sutures, and each animal received 5 mL of pre-warmed 0.9% saline (subcutaneous) and tramadol (5 mg/kg, subcutaneous), with analgesia maintained every 12 h. No antibiotics were administered, consistent with the standard protocol applied in our laboratory for this CLP severity model.

After 48 h, the animals (n = 17) underwent a second echocardiographic assessment and were subsequently euthanized.

#### 2.1.2. Cardiac Changes in the 7-Day Subacute Follow-Up

**Figure 1:**
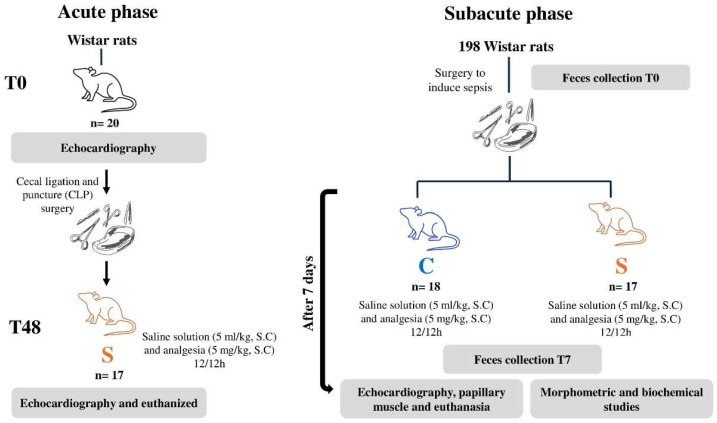
**Experimental design.** C, control; S, sepsis; S.C, subcutaneous; T0, stool collection at baseline; T7, stool collection at time on day 7.

Male Wistar rats (n = 198), aged 8 weeks and weighing 250–300 g, were used in this phase. After an acclimatization period, the animals were randomly assigned into two experimental groups: control (sham, C) and sepsis (S) (Figure 1). The surgical procedures were similar to those performed in the acute phase, except that cecal perforation was not performed in control animals. Fecal samples from six rats per group were collected before the procedure and 7 days afterward.

The animals were monitored daily for survival, clinical signs of sepsis, food intake, and body weight. Seven days after surgery, the animals underwent echocardiographic assessment, followed by euthanasia, papillary muscle testing, and subsequent morphometric and biochemical analyses.

### 2.2. Echocardiography

The animals were anesthetized with ketamine (50 mg/kg, intraperitoneal [i.p]) and xylazine (1 mg/kg, i.p.) and positioned in the left lateral decubitus position. Echocardiographic examinations were performed using a Vivid S6 system (GE Medical Systems, Milwaukee, WI, USA) equipped with a multifrequency transducer (5–11.5 MHz). All measurements followed established methodological guidelines for rodent echocardiography, based on protocols validated for use in rats [15,16].

Echocardiography was conducted at baseline and 48 h post-sepsis induction to evaluate acute-phase cardiac dysfunction. After seven days, LV structural parameters were obtained, including diastolic diameter (LVDD), systolic diameter, posterior wall thickness, and interventricular septal thickness, using M-mode imaging. Left atrium (LA) and aortic root (AO) dimensions were measured in the parasternal long-axis view, and the LA/AO ratio was used as a marker of atrial remodeling. Additionally, LV mass (calculated using a validated formula), LV mass index (normalized by body weight), relative wall thickness, and body weight-adjusted values (LVDD/BW and LA/BW) were determined.

Cardiac function was assessed using systolic and diastolic parameters obtained via M-mode, pulsed-wave, and tissue Doppler echocardiography [8]. For systolic function, the following parameters were analyzed: EF, average systolic velocity of the mitral annulus (S’, cm/s), posterior wall shortening velocity (PWSV, mm/s), endocardial fractional shortening (EFS), and myocardial performance index (Tei index), calculated as (IVRT + IVCT)/ejection time, where IVRT is isovolumic relaxation time and IVCT is isovolumic contraction time. For diastolic function, the following parameters were evaluated: E/A ratio (transmitral flow, apical four-chamber view), IVRT, and the IVRT/R-R ratio (adjusted for heart rate).

### 2.3. Myocardial Function Assessed by Papillary Muscle

Functional studies of isolated LV papillary muscles were performed following standardized techniques described in previous studies [17,18]. LV papillary muscles were dissected and mounted in Krebs–Henseleit solution (28 °C, pH 7.38–7.42, 95% O_2_/5% CO_2_). Force was recorded using a Kyowa transducer, and length was adjusted via a lever system. Muscles were stimulated at 12/min with 5 ms pulses, at 10% above the stimulation threshold. After 60 min of isotonic contractions, preparations were switched to isometric mode and stretched to their optimal length. Following 15 min of stable contractions, a single twitch was recorded. The following parameters were analyzed: developed tension, resting tension, +dT/dt, −dT/dt, and time to peak tension.

Contractile function of the papillary muscles was assessed under basal conditions (extracellular Ca^2+^ 2.5 mM). All variables were normalized to cross-sectional area (CSA = weight/Lmax; density = 1), and only muscles with CSA between 0.5 and 1.5 mm^2^ and performance within ±1.96 SD were included.

### 2.4. Euthanasia and Sample Collection

Animals were euthanized with a single dose of thiopental (120 mg/kg, i.p.). The hearts were dissected, and the right ventricle and LV, including the interventricular septum, were separated and weighed. Basal and apical regions of the LV were stored at −80 °C for subsequent analyses.

### 2.5. Histology

The LV was sectioned 4 mm from the apex. Tissue fragments were fixed in 10% formalin for 24 h, transferred to 70% ethanol, embedded in paraffin, and sectioned for histological analysis [19]. Slides were stained with hematoxylin and eosin (HE) to access cardiomyocyte CSA and with picrosirius red for collagen quantification. Images were captured using a 40× objective under polarized light microscopy (LEICA DMLB, DFC 295 camera, LAS 4.2 software) [20]. Ten images per slide were analyzed, with CSA measured in 15 cardiomyocytes per field. Collagen content was quantified by calculating the mean fluorescence intensity from 10 fields using ImageJ software 1.54g (National Institutes of Health, Bethesda, MD, USA).

### 2.6. Cardiac Energy Metabolism

LV samples (30 mg) were homogenized in 300 μL of buffer (50 mM Tris-HCl, 1 mM EDTA, 0.1% Triton X-100, protease inhibitor, pH 7.4) using 0.5 mm glass beads in a Bullet Blender^®^ homogenizer (Next Advance, Troy, NY, USA) at 12,000 rpm for 5 min at 4 °C. The homogenate was centrifuged at 4000 rpm for 10 min at 4 °C, and the supernatant was used for further analyses. The maximal activity of the following energy metabolism enzymes was assessed in duplicate at 25 °C: hexokinase, phosphofructokinase, pyruvate kinase, aldolase, lactate dehydrogenase, carnitine palmitoyl transferase I, and creatine kinase. Enzymatic reactions were monitored by spectrophotometry in microplates (Agilent BioTek Epoch, Winooski, VT, USA), with readings taken every 60 s for 10 min at 340 nm or 412 nm, depending on the assay. Blanks were run using buffer without sample. Protein concentration was determined using the Bradford method [21]. Enzymatic assays were performed with specific buffers for each enzyme, containing appropriate substrates and cofactors such as Tris-HCl, MgSO_4_, NADH, ATP, fructose-6-phosphate, phosphoenolpyruvate, oxaloacetate, palmitoyl-CoA, and creatine, according to standardized protocols. Reactions were initiated by adding the specific substrate for each enzyme.

### 2.7. Oxidative Stress Biomarkers

LV samples (50 mg) were homogenized in 500 μL of 1.15% KCl buffer. The homogenate was centrifuged, and the supernatant was collected for analysis. Total protein concentration was determined as previously described [22]. Protein carbonylation was measured by incubating 100 μL of supernatant with 100 μL of DNPH (10 mM in 2 M HCl), followed by the addition of 50 μL of NaOH (6 M) and a further 10 min incubation. Absorbance was measured at 450 nm using a microplate reader (Spectra Max 190, Molecular Devices, San José, CA, USA), and results were expressed as nmol carbonyl/mg protein using a molar extinction coefficient of 22,000 M^−1^ cm^−1^. Malondialdehyde levels were measured using the thiobarbituric acid reactive substances method. For quantification, 200 µL of supernatant from the homogenate of each sample was diluted in 500 µL of solution containing 0.67% thiobarbituric acid, 10% trichloroacetic acid, and 0.25 M HCl, then centrifuged. Samples were incubated in a water bath at approximately 100 °C for 45 min and subsequently transferred to a microplate (200 µL). Absorbance was recorded at 532 nm and 600 nm on a Spectra Max 190 microplate reader (Molecular Devices^®^, Sunnyvale, CA, USA). Superoxide dismutase (SOD) activity was determined based on inhibition of pyrogallol auto-oxidation using Tris-EDTA buffer and pyrogallol, with spectrophotometric reading at 420 nm. Results were expressed as SOD units/mg protein. Catalase activity was assessed by monitoring hydrogen peroxide (H_2_O_2_) consumption through the decrease in absorbance at 240 nm. Reactions were carried out in 50 mM sodium phosphate buffer containing 1 mM EDTA (pH 7.0) and 0.3 mol/L H_2_O_2_. Results were expressed as mol H_2_O_2_ consumed/mg protein.

### 2.8. Metagenomic (Microbiota Analysis)

Fecal samples from six animals per group were collected at baseline and after 7 days for microbiota analysis. During each collection, rats were placed individually in clean, bedding-free containers, and freshly voided pellets were transferred into cryogenic tubes and stored at −80 °C until DNA extraction and sequencing. Genomic DNA was extracted from fecal samples using the QIAamp DNA Stool Mini Kit (Qiagen, Valencia, CA, USA) following the manufacturer’s instructions. The V3–V4 hypervariable regions of the bacterial 16S rRNA gene were amplified using primers tailed with Illumina adapter overhangs (Forward: 5′-TCGTCGGCAGCGTCAGATGTGTATAAGAGACAGCCTACGGGNGGCWGCAG-3′; Reverse: 5′-GTCTCGTGGGCTCGGAGATGTGTATAAGAGACAGGACTACHVGGGTATCTAATCC-3′) [23]. PCR was performed in 25 µL reactions containing 12.5 µL GoTaq^®^ Green Master Mix (Promega, Fitchburg, WI, USA), 5 µL of each primer (1 µM), and 2.5 µL DNA template (5 ng/µL). Thermal cycling consisted of an initial denaturation at 95 °C for 3 min; 25 cycles of 95 °C for 30 s, 55 °C for 30 s, 72 °C for 30 s; and a final extension at 72 °C for 5 min.

Amplicons were purified with AMPure XP beads (Beckman Coulter, Brea, CA, USA) at a 0.8× ratio, then dual-indexed using the Nextera XT Index Kit (Illumina, San Diego, CA, USA) according to the manufacturer’s protocol. Indexed libraries were quantified by qPCR using the NEBNext^®^ Library Quant Kit for Illumina (New England Biolabs, Ipswich, MA, USA), normalized to 4 nM, and pooled.

### 2.9. Statistical Analysis

Data normality was assessed using the Shapiro–Wilk test. Variables with a normal distribution are expressed as mean ± standard deviation (SD), whereas variables with a non-normal distribution are presented as median (including the lower quartile and upper quartile). In the acute phase, a paired *t*-test was used. In the chronic phase, comparisons between groups were performed using Student’s *t*-test or the nonparametric Mann–Whitney U test, as appropriate. All analyses were conducted using Jamovi software version 2.3 (https://www.jamovi.org).

For gut microbiota analysis, FASTQ files containing 300 base pair (bp) paired-end reads were processed using Quantitative Insights Into Microbial Ecology (QIIME, version 1.8.0). Sequences were overlapped and filtered for quality. Operational taxonomic units were clustered at 97% similarity using the UCLUST algorithm. Diversity analysis included rarity curves and UniFrac metrics to obtain distance matrices with a depth of 600α, as well as principal component analysis. The significance of differences at baseline and after 7 days of follow-up was tested using permutational multivariate analysis of variance (PERMANOVA, with *p* < 0.05). Taxonomic abundance was expressed as percentages. A significance level of *p* ≤ 0.05 was adopted.

## 3. Results

### 3.1. Acute Phase

The 48 h mortality in sepsis was 15%. Echocardiographic assessment revealed that, following sepsis, animals exhibited reduced BW, EFS, PWSV, and EF, whereas heart rate (HR), LA, LA/AO, and LA/BW were increased (Table 1).

### 3.2. Cardiac Changes in the 7-Day Subacute Follow-Up

#### 3.2.1. Experimental Groups and Anatomical Parameters

After 7 days, the mortality rate among animals subjected to sepsis was 90.5%. Septic animals exhibited a reduction in BW compared with the control group (269 ± 30.1 vs. 294 ± 21.0; *p* = 0.009). Furthermore, relative organ weights, calculated as the ratio of organ weight to body weight, for the kidney (*p* = 0.001), liver (*p* < 0.001), and spleen (*p* < 0.001) were significantly higher in the septic group than in the control group, as shown in Table 2.

#### 3.2.2. Echocardiogram

Structural and functional echocardiographic variables measured after 7 days are presented in Table 3 and Table 4. LVDD/BW (*p* = 0.012) and LA/BW (*p* = 0.011) were higher in septic animals compared with that in controls. HR [402.00 (385.50–417.50) vs. 433.50 (399.75–450.00); *p* = 0.035] and PWSV (45.05 ± 6.06 vs. 50.54 ± 6.06; *p* = 0.027) were lower in septic animals than in controls.

#### 3.2.3. Myocardial Function Assessed by Papillary Muscle

Analysis of papillary muscles revealed that the time to develop tension (DT) was shorter in the sepsis group, whereas relaxation time (RT) and time to peak tension were prolonged. Moreover, the maximum rate of tension development (+dT/dt) was reduced in the sepsis group compared with that in the control group (Table 5).

#### 3.2.4. Histology

**Figure 2:**
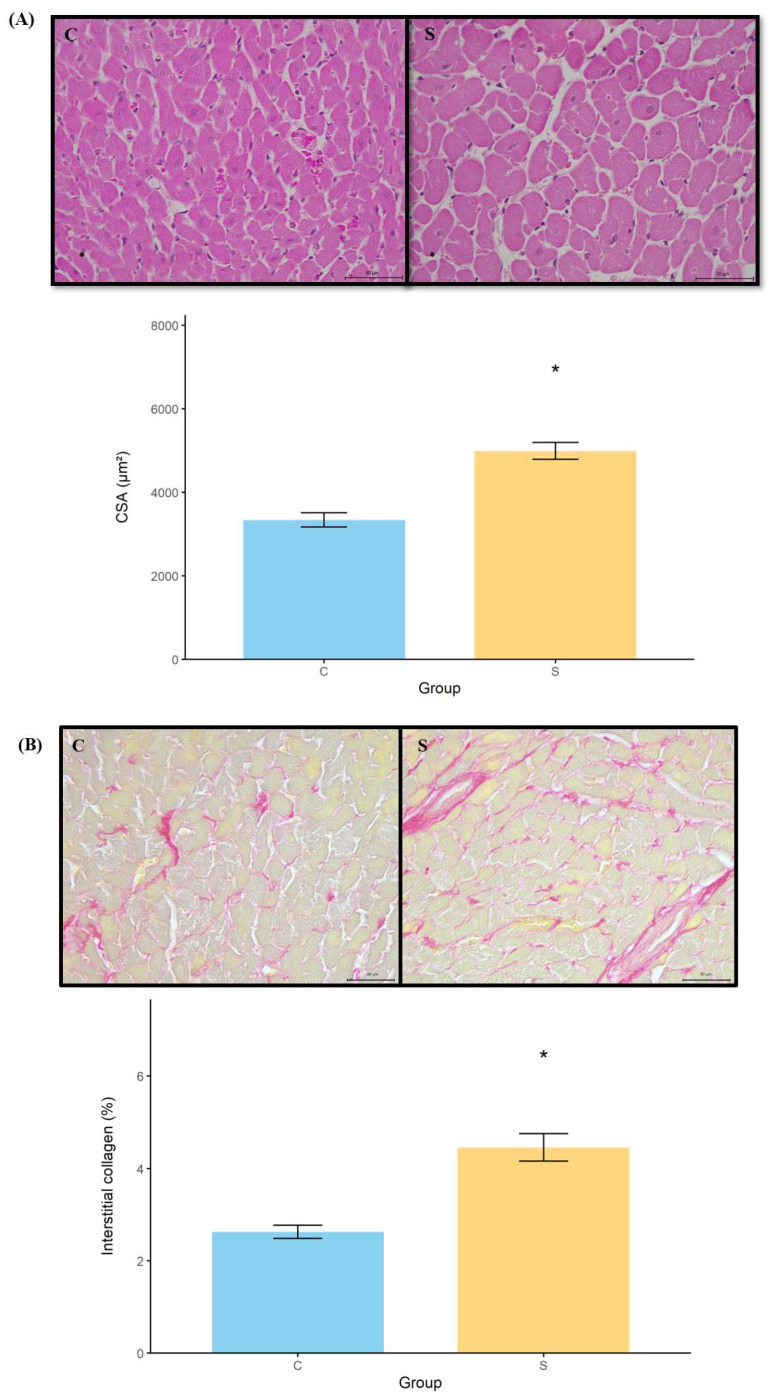
**Histological analysis of cardiac tissue using hematoxylin–eosin and picrosirius red staining**. (**A**) CSA: Cardiomyocyte cross-sectional area; (**B**) percentage of interstitial collagen fraction. * *p* < 0.05 vs. control group. Student’s *t*-test. C (n = 9), control; S (n = 11), sepsis.

Cardiomyocyte CSA and collagen content were significantly greater in the sepsis group compared with that in the control group, as illustrated in Figure 2.

#### 3.2.5. Cardiac Energy Metabolism

Analysis of maximal enzymatic activities involved in cardiac energy metabolism revealed no significant differences between groups (Table 6).

#### 3.2.6. Oxidative Stress Biomarkers

The expression of oxidative damage markers and the activities of antioxidant enzymes did not differ between groups. The data are summarized in Table 7.

#### 3.2.7. Metagenomic Analysis

No differences were observed in gut microbiota at baseline, confirming homogeneity between groups before the start of the experimental protocol. Analysis of samples collected 7 days after sepsis induction showed that rarefaction curves reached a plateau, indicating sufficient sequencing depth to capture most microbial diversity. Alpha diversity, assessed by evenness, did not differ between time points. In contrast, beta diversity analysis of relative data revealed significant differences between septic animals and controls (Table 8).

**Figure 3:**
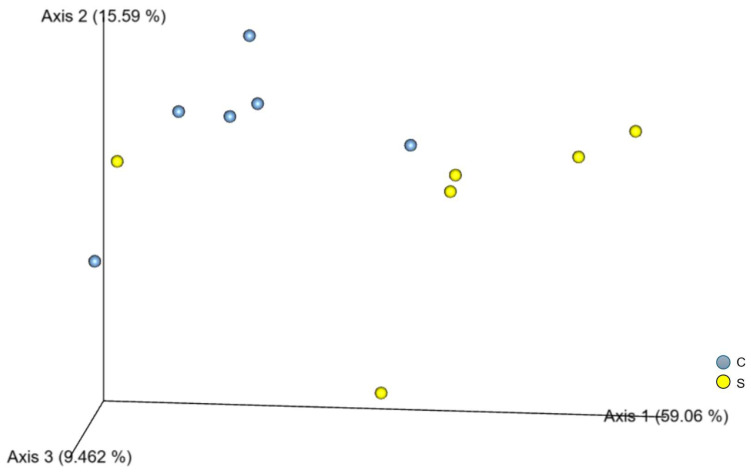
Principal component analysis of the gut microbiota from samples collected at the end of treatment. C (n = 6), control; S (n = 6), sepsis.

**Figure 4:**
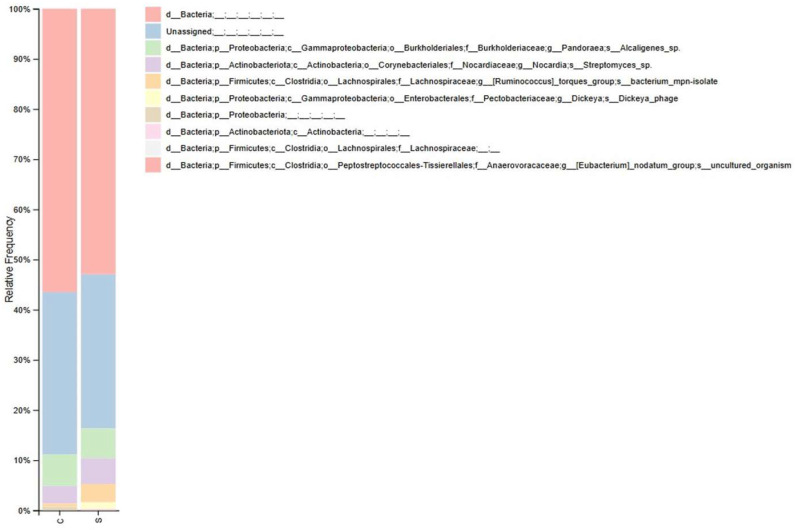
Taxonomic classification of gut microbiota. C (n = 6), control; S (n = 6), sepsis.

Principal component analyses for both absolute and relative data confirmed significant differences in microbial community structure between groups (Figure 3). However, taxonomic classification at the phylum, class, order, and family levels showed no changes in the relative abundance of major bacterial taxa between groups (Figure 4).

## 4. Discussion

Despite growing interest in septic cardiomyopathy, many aspects of its pathophysiology remain understood. A critical consideration is that septic insult to the heart may induce latent changes characteristic of cardiac remodeling, with future consequences. The present study aimed to evaluate the morphological and functional cardiac changes in the acute and subacute phases (with 7-day follow-up) in male Wistar rats subjected to experimental sepsis using a cecal ligation and puncture (CLP) model. Our findings suggest that in this experimental context, septic cardiomyopathy may exhibit functional and structural alterations that persist beyond the acute phase, indicating that recovery may not be fully complete by day 7.

Some patients with sepsis had reported reversible depression in LV systolic function and ventricular dilation. These characteristics continue to be used to describe sepsis cardiomyopathy. Over time, the definition of this condition has expanded to include other cardiac abnormalities, such as LV diastolic disfunction and right ventricular failure [24].

In the acute phase, sepsis induced both systolic and diastolic dysfunction, consistent with observations in humans and characteristic of septic cardiomyopathy [8,9,10,11]. Although no formal definition exists, sepsis-induced myocardial dysfunction is generally considered an acute process that is reversible after some days [9,10]. Recent studies using cardiac magnetic resonance imaging have provided additional insights into the potential reversibility of this condition [11].

In the 7-day period following the induction of sepsis, histological analysis revealed initial signs of myocyte hypertrophy and increased collagen deposition in the myocardium. Although cardiac remodeling is a highly complex process, the presence of hypertrophy and fibrosis is critical in characterizing remodeling [25]. Furthermore, collagen accumulation appears to be a more permanent event, with a lower likelihood of remission. Notably, the collagen accumulation occurred predominantly as type III collagen, similar to patterns observed in cardiac remodeling following myocardial infarction. Considering the implications of our findings, in different models of cardiac remodeling, fibrosis was associated with increased myocardial stiffness, diastolic dysfunction, weakened contraction, impaired coronary flow and malignant arrhythmias. In addition, fibrosis was a predictor of mortality in patients with cardiac dysfunction [25,26,27].

Considering functional aspects, systolic dysfunction assessed via echocardiography persisted seven days after sepsis induction. This finding was confirmed by studies using isolated papillary muscle. Experiments with isolated cardiac muscle allow assessment of myocardial mechanical function without the influence of extrinsic and confounding factors, such as coronary circulation, neurohormonal system, and changes in loading conditions [28,29,30], better assessing the intrinsic functional capacity of the heart. In fact, our findings suggest that the intrinsic ability of the muscle to contract and relax was compromised after 7 days of sepsis. Therefore, these results indicate that sepsis induces LV systolic and diastolic dysfunction, which remains evident in the subacute phase. Therefore, our study showed that after 7 days, sepsis induced myocyte hypertrophy, collagen accumulation, and persistent ventricular dysfunction. These variables are closely related and are critical markers of the cardiac remodeling process in different models.

Sepsis is also associated with long-term complications among survivors. The underlying mechanisms are not fully understood but appear to be multifactorial, including cognitive impairment, functional impairment, and new or worsening chronic health conditions [6]. These findings suggest that cardiac septic remodeling may represent an additional mechanism contributing to cardiovascular events in sepsis survivors and could have potential as a therapeutic target in this clinical setting. In support of our results, in other clinical scenarios, including COVID-19 infection or Takotsubo Syndrome, emerging evidence has already challenged the traditional view that myocardial injury is solely an acute and reversible condition [31,32].

The potential pathophysiological mechanisms underlying cardiac remodeling are multifactorial, with the contribution of each factor appearing to depend on the model of cardiac injury [33,34,35,36]. In the 7-day interval following sepsis induction, no changes were observed in energy substrate proteins, or redox balance. However, in the context of this study, these findings should be interpreted with caution, suggesting that the absence of measurable changes at this time point does not necessarily imply the absence of participation of these mechanisms in the cardiac remodeling process. It is important to recognize that the biochemical analyses were conducted with relatively small sample sizes and evaluated only at a late point in the evolution of sepsis. Thus, it is possible that mitochondrial, or oxidative changes occurred predominantly in the acute phase (24–72 h) and were attenuated or normalized by the seventh day.

Recent studies have identified an association between heart failure and the gut microbiota. Heart failure, through mechanisms such as low perfusion, systemic congestion, or the release of mediators, may alter the gut microbiota composition, compromising the intestinal barrier and increased permeability. This process facilitates the translocation of microorganisms and mediators, exacerbating inflammation, a known modulator of cardiac remodeling [37]. In the present study, gut microbiota analysis reveals differences in β-diversity between groups, without significant changes in microbial abundance at the phylum, class, order, or family levels. Therefore, the study cannot establish a mechanistic link between microbiota alterations and cardiac remodeling but suggests their association. Thus, the exact role of the microbiota as a modulator of the remodeling process in this model remains to be elucidated.

Our results should be interpreted taking into account potential limitations. The mortality rate during the 7-day follow-up reaches 90%. Therefore, the final analyses are based only on a small subset of surviving animals, which are highly likely to represent a milder phenotype or a resilient subpopulation with greater tolerance to septic injury. This could introduce potential bias and make it difficult to generalize our results.

## 5. Conclusions

After seven days, sepsis-induced cardiac remodeling is characterized by persistent systolic and diastolic dysfunction, accompanied by cardiomyocyte hypertrophy and fibrosis. In this model of CLP in Wistar rats, septic cardiomyopathy should not be considered merely an acute, transient, and reversible condition.

## Figures and Tables

**Table 1:** Echocardiographic parameters 48 h after sepsis induction.

Variable	T0 (n = 20)	T48 (n = 18)	*p*-Value
BW (g)	209.35 ± 10.85	196.22 ± 14.98	**0.004**
HR (bpm)	400.85 ± 37.88	439.67 ± 39.76	**0.004**
LVDD (mm)	6.73 ± 0.39	6.49 ± 0.45	0.085
LVSD (mm)	3.00 ± 0.52	3.32 ± 0.79	0.151
PWT (mm)	1.09 ± 0.04	1.09 ± 0.03	0.996
IVST (mm)	1.10 ± 0.04	1.10 ± 0.02	0.795
AO (mm)	3.25 ± 0.09	3.19 ± 0.11	0.081
LA (mm)	4.67 (4.53–4.83)	5.04 (4.82–5.26)	**<0.001**
LA/AO	1.42 (1.38–1.49)	1.57 (1.52–1.64)	**<0.001**
LVDD/BW (mm/kg)	32.26 ± 2.93	33.22 ± 3.00	0.324
LA/BW (mm/kg)	22.40 (20.85–23.81)	25.39 (24.10–27.89)	**<0.001**
EFS (%)	55.60 ± 6.19	49.29 ± 9.82	**0.022**
LV mass (g)	0.42 (0.40–0.43)	0.39 (0.37–0.42)	0.087
LVMI (g/m^2^)	1.98 (1.82–2.14)	2.09 (1.93–2.16)	0.492
RWT	0.32 ± 0.02	0.34 ± 0.03	0.094
PWSV (cm/s)	43.19 ± 5.66	38.09 ± 8.16	**0.030**
IVRT/R-R (ms)	21.00 (18.00–23.50)	18.00 (18.00–20.50)	0.103
EF (%)	0.90 (0.88–0.94)	0.87 (0.84–0.91)	**0.022**

T0, baseline; T48, after 48 h of sepsis; BW, body weight; HR, heart rate; LVDD, left ventricular diastolic diameter; LVSD, left ventricular systolic diameter; PWT, left ventricular posterior wall diastolic thickness; IVST, intraventricular septal diastolic.

**Table 2:** Body and organ weights 7 days after sepsis induction.

Variable	C (n = 18)	S (n = 17)	*p*-Value
BW (g)	294 ± 21.0	269 ± 30.1	**0.009**
LV (g)	0.5 ± 0.1	0.5 ± 0.1	0.394
LV/BW (g/kg)	1.8 (1.2–2.2)	1.8 (1.6–2.4)	0.496
RV (g)	0.2 ± 0.0	0.2 ± 0.0	0.755
RV/BW (g/kg)	0.6 ± 0.1	0.6 ± 0.1	0.100
Kidney (g)	1.0 (0.9–1.2)	1.1 (0.9–2.9)	0.064
Kidney/BW (g/kg)	3.5 (3.1–4.3)	4.2 (3.3–13.2)	**0.001**
Lung (g)	1.3 ± 0.2	1.3 ± 0.3	0.834
Lung/BW (g/kg)	4.3 (3.5–5.3)	4.3 (3.8–8.2)	0.423
Liver (g)	10.5 ± 1.3	10.8 ± 1.5	0.587
Liver/BW (g/kg)	35.9 ± 3.8	40.5 ± 6.7	**0.019**
Spleen (g)	0.7 ± 0.1	1.0 ± 0.2	**<0.001**
Spleen/BW (g/kg)	2.4 ± 0.4	3.9 ± 1.1	**<0.001**

C, control; S, sepsis; BW, body weight; LV, left ventricle; RV, right ventricle. Data are expressed as mean and standard deviation or median (including the lower and upper quartile).

**Table 3:** Body weight and structural echocardiographic parameters 7 days after sepsis induction.

Variable	C (n = 14)	S (n = 13)	*p*-Value
BW (g)	292.36 ± 19.67	265.69 ± 31.02	**0.013**
LVDD (mm)	6.78 ± 0.43	6.79 ± 0.37	0.870
LVSD (mm)	3.01 ± 0.48	3.10 ± 0.38	0.576
PWT (mm)	1.22 (1.18–1.26)	1.19 (1.16–1.23)	0.152
IVST (mm)	1.22 (1.18–1.26)	1.19 (1.16–1.23)	0.152
AO (mm)	3.64 ± 0.27	3.62 ± 0.28	0.864
LA (mm)	4.83 ± 0.30	5.06 ± 0.46	0.151
LA/AO	1.33 ± 0.11	1.39 ± 0.11	0.135
LVDD/BW (mm/kg)	23.21 (23.56–23.90)	24.88 (23.52–28.53)	**0.012**
LA/BW (mm/kg)	16.49 (15.42–17.44)	19.04 (16.53–21.78)	**0.011**
LVM (g)	0.49 ± 0.07	0.48 ± 0.06	0.517
LVMI (g/kg)	1.73 (1.62–1.76)	1.79 (1.59–1.94)	0.182
RWT	0.36 (0.02–0.00)	0.35 (0.02–0.00)	0.215

C, control; S, sepsis; BW, body weight; LVDD, left ventricular diastolic diameter; LVSD, left ventricular systolic diameter; PWT, left ventricular posterior wall diastolic thickness; IVST, intraventricular septal diastolic thickness; AO, aorta diameter; LA, left atrial diameter; LVM, left ventricular mass; LVMI, left ventricular mass index; RWT, relative wall thickness. Data are expressed as mean and standard deviation or median (including the lower and upper quartile).

**Table 4:** Functional echocardiographic parameters 7 days after sepsis induction.

Variable	C (n = 14)	S (n = 13)	*p*-Value
HR (bpm)	433.50 (399.75–450.00)	402.00 (385.50–417.50)	**0.035**
EFS (%)	55.72 ± 5.53	54.42 ± 4.31	0.506
PWSV (mm/s)	50.54 ± 6.06	45.05 ± 6.06	**0.027**
IVRT/R-R (ms)	52.81 ± 5.53	50.09 ± 7.04	0.275
TEI index	0.41 ± 0.05	0.44 ± 0.03	0.069
EF (%)	0.91 ± 0.03	0.91 ± 0.02	0.587
S’mean (cm/s)	3.88 ± 0.22	3.97 ± 0.26	0.341

C, control; S, sepsis; HR, heart rate; EFS, endocardial fractional shortening; PWSV, posterior wall shortening velocity; IVRT/R-R, LV isovolumetric relaxation time; TEI index, myocardial performance index; S’ mean, mean of lateral and septal mitral annulus velocity in systole. Data are expressed as mean and standard deviation or median (including the lower and upper quartile).

**Table 5:** In vitro functional assessment of left ventricular papillary muscle.

Variable	C (n = 7)	S (n = 8)	*p*-Value
DT (g/mm^2^)	7.3 ± 0.5	6.0 ± 0.8	**0.003**
RT (g/mm^2^)	0.7 ± 0.1	1.0 ± 0.3	**0.031**
TPT (ms)	175 (160–215)	198 (185–220)	**0.013**
+dT (g/mm^2^)	82.6 ± 7.0	66.3 ± 6.6	**<0.001**
−dT (g/mm^2^)	22.5 ± 3.9	21.6 ± 3.7	0.636
CSA (mm^2^)	0.9 (0.7–1.2)	0.9 (0.8–1.4)	0.867

C, control; S, sepsis; DT, developed tension (g/mm^2^); RT, resting tension (g/mm^2^); TPT, time to reach the maximum peak of the developed tension (ms); +dT/dt, maximum rate of increase in the developed tension (g/mm^2^/s); −dT/dt, maximum rate of decrease in the developed tension (g/mm^2^/s); CSA, cross-sectional area (mm^2^). Data are expressed as mean ± standard deviation or median (including the lower and upper quartile).

**Table 6:** Maximal enzymatic activities related to cardiac energy metabolism.

Variable	C (n = 10)	S (n = 11)	*p*-Value
Hexokinase	0.007 (0.006–0.010)	0.008 (0.005–0.012)	0.173
Phosfofructokinase	0.007 ± 0.001	0.008 ± 0.002	0.416
Pyruvate kinase	0.072 ± 0.017	0.085 ± 0.081	0.233
Aldolase	0.002 (−0.001–0.007)	0.002 (−0.001–0.004)	0.654
LDH	0.109 (0.087–0.185)	0.128 (0.089–0.144)	0.223
CPT-1	5.06 ± 2.16	6.25 ± 1.70	0.175
Creatine kinase	0.010 ± 0.003	0.010 ± 0.004	0.709

C, control; S, sepsis; LDH, lactate dehydrogenase; CPT-1, carnitine palmitoyl transferase 1. Data are expressed as mean ± standard deviation or median (including the lower and upper quartile). Enzymatic activity was expressed as µmol of substrate converted per minute per mg of protein (µmol/min·mg protein).

**Table 7:** Oxidative stress biomarkers.

Variable	C (n = 5)	S (n = 6)	*p*-Value
Protein carbonylation	6.3 ± 1.1	6.2 ± 1.1	0.945
MDA	38.4 ± 10.4	32.8 ± 13.1	0.459
SOD	4.8 ± 0.6	4.7 ± 0.4	0.743
CAT	1.3 ± 0.2	1.6 ± 0.5	0.212

C, control; S, sepsis; SOD, superoxide dismutase; CAT, catalase; MDA, malondialdehyde. Data are expressed as mean and standard deviation. Data were expressed as µmol of substrate converted per minute per mg of protein (µmol/min·mg protein).

**Table 8:** Relative β-diversity of gut microbiota.

Group	Comparison	H	*p*-Value
C (n = 6)	S (n = 6)	3.507	**0.031**

C, control; S, sepsis.

## Data Availability

The data presented in the study are openly available in the UNESP Institutional Repository at https://hdl.handle.net/11449/320440 (accessed on 5 March 2026).
